# Network Pharmacology to Unveil the Mechanism of Berberine in the Treatment of *Streptococcus suis* Meningitis in Humans and Pigs

**DOI:** 10.3390/toxics13020138

**Published:** 2025-02-15

**Authors:** Pu Guo, Yunda Xue, Dan Zhang, Qirong Lu, Yu Liu, Jianglin Xiong, Chun Ye, Shulin Fu, Zhongyuan Wu, Xu Wang, Yinsheng Qiu

**Affiliations:** 1 Hubei Key Laboratory of Animal Nutrition and Feed Science, School of Animal Science and Nutritional Engineering, Wuhan Polytechnic University, Wuhan 430023, China; guopu@whpu.edu.cn (P.G.); xue18337292680@163.com (Y.X.); 15823132897@163.com (D.Z.); qirongluvet@whpu.edu.cn (Q.L.); lyywfy@whpu.edu.cn (Y.L.); xiongjianglin@126.com (J.X.); yechun@whpu.edu.cn (C.Y.); shulinfu@whpu.edu.cn (S.F.); zhongywu@whpu.edu.cn (Z.W.); 2 Wuhan Engineering and Technology Research Center of Animal Disease-Resistant Nutrition, School of Animal Science and Nutritional Engineering, Wuhan Polytechnic University, Wuhan 430023, China; 3 National Reference Laboratory of Veterinary Drug Residues (HZAU) and MAO Key Laboratory for Detection of Veterinary Drug Residues, Huazhong Agricultural University, Wuhan 430070, China

**Keywords:** berberine, *S. suis*, meningitis, anti-inflammatory, anti-oxidative stress, network pharmacology

## Abstract

*Streptococcus suis* (*S. suis*) is a major swine pathogen throughout the world as well as an emerging zoonotic agent. Among the symptoms caused by *S. suis*, including septicemia, pneumonia, endo-carditis, arthritis, and meningitis, the latter is the most overlooked. In the present study, we explored the mechanism of action of berberine against *S. suis* meningitis by obtaining berberine-related action targets, porcine *S. suis* meningitis targets, and human *S. suis* meningitis targets from open databases. We constructed a protein–protein interaction (PPI) network by using the STRING database and employed Cytoscape 3.8.0 to screen for core targets. We performed Gene Ontology (GO) and Kyoto Encyclopedia of Genes and Genomes (KEGG) enrichment analyses through DAVID. We identified 31 potential targets of berberine, of which Toll-like receptor 4 (TLR4), fibronectin 1 (FN1), superoxide dismutase (SOD1), and catalase (CAT) were the four most critical targets. GO analysis revealed the enrichment of terms related to the response to oxidative stress and the inflammatory response. KEGG analysis revealed the enrichment of the interleukin 17 (IL-17), phosphoinositide 3-kinase (PI3K)-Akt, TLR, tumor necrosis factor (TNF), and mitogen-activated protein kinase (MAPK) signaling pathways. In addition, the admetSAR results showed that berberine can cross the blood–brain barrier. The molecular docking results indicated key binding activity between TLR4–berberine and FN1–berberine. In summary, berberine protects against *Streptococcus suis* meningitis by regulating inflammatory response and oxidative stress in humans and pigs. Our study updates the current knowledge of the targets of *S. suis* meningitis to exploit new drugs in humans and pigs, to develop environmentally friendly and antibiotic-free animal-derived food products, and to improve the farming industry and economic development.

## 1. Introduction

*Streptococcus suis* (*S. suis*), a Gram-positive bacterium, is a major swine pathogen and causes considerable economic losses in the swine industry [1]. As an important zoonotic pathogen, *S. suis* can cause septicemia, pneumonia, endocarditis, arthritis, and meningitis, with irreversible sequelae in pigs and humans [2,3]. Meningitis, a symptom that is often overlooked, has begun to receive attention in recent years due to the increasing number of reported cases. In one study, *S. suis* was isolated from 95% of the sampled pigs in both the case (previously diagnosed *S. suis* infections) and control (clinically healthy Swedish pigs) herds. Moreover, 8 of the 10 case herds showed clinical signs indicative of meningitis [4]. *S. suis* infection not only sharply reduces the economic benefits of pig farming but also has severe effects on humans. It can be transmitted to humans by eating undercooked pork or by slaughtering sick pigs.

The number of *S. suis* infections in humans has increased substantially. From July to August 2005, there has been a large-scale public health crisis in Sichuan due to *S. suis* infection. A total of 215 cases of *S. suis* infection were reported, resulting in 38 deaths, and 48% of the infected patients had meningitis [5]. Recently, Hospital Universitario de Canarias presented a case report of meningitis with associated secondary bacteremia due to *S. suis* infection in a 56-year-old man [6]. In a case-based surveillance for bacterial meningitis in Burkina Faso, from 2015 to 2018, there were 38 cases of meningitis among 590 people who were polymerase chain reaction (PCR) positive for three nonpneumococcal streptococcal pathogens; 21 of these cases involved *S. suis* infection. The 21 *S. suis*-positive patients were of different ages, the majority were male (16, 76.2%), and 2 (9.5%) patients died [7]. In an Indonesian study, of 71 acute bacterial meningitis cases, *S. suis* was confirmed in the cerebrospinal fluid culture of 44 patients with an average age of 48.1 years; 89% of the patients were male [8]. Two studies in Thailand showed that *S. suis* infections in humans are responsible for an estimated loss in productivity-adjusted life years to the gross domestic product of USD 11.3 million, which equals USD 36,033 lost per person and USD 140 out-of-pocket expenses for each patient [9,10].

At present, the target(s) of *S. suis* meningitis in pigs and humans and its pathogenic mechanism remain unknown, meaning that there are great obstacles to the treatment of the disease and the creation of novel anti-streptococcal meningitis drugs. The search for highly effective preventive and therapeutic drugs with few side effects will help to strengthen the prevention of this disease, reduce the risk of infection and transmission among practitioners and the morbidity and mortality of porcine and human meningitis, improve the efficiency of farming, and contribute to modernizing the swine industry and disease prevention and control.

Berberine is a plant alkaloid found in the roots and bark of a variety of plants, including Coptis chinensis (goldenthread), Berberis aquifolium (Oregon grape), Berberis vulgaris (barberry), Hydrastis canadensis (goldenseal), and Berberis aristata (tree turmeric). Berberine exhibits a wide range of pharmacological effects, including anti-inflammatory, antioxidant, immunomodulatory, anti-tumor, and antibacterial activities [11,12,13,14]. Berberine has a good bacteriostatic effect on *S. suis*, with a minimum inhibitory concentration (MIC) of 0.19 mM [15]. Berberine could reduce cerebral hemorrhage-induced neurological deficits and inflammatory cytokine production, inhibit inflammatory responses, and protect the integrity of the blood–brain barrier in a mouse cerebral hemorrhage brain injury model established by injecting autologous whole blood [16]. Moreover, berberine could effectively reduce doxorubicin-induced neuroinflammation and oxidative stress, suggesting that this compound exerts a favorable neuroprotective effect through its antioxidant and anti-inflammatory activities at the level of nerve inflammasome [17]. Furthermore, in a rat model of chronic cerebral hypoperfusion, berberine hydrochloride restored chronic cerebral hypoperfusion-induced neuronal damage and counteracted cognitive impairment [18], indicating that berberine may be useful to treat neuroinflammation and neurodegenerative disorders.

The active ingredients in traditional Chinese medicine can act through various targets and have complex routes of action. Given that the mechanism of action of berberine against *S. suis* meningitis remains unclear, we constructed a component–disease pathway–target network to analyze the relevant targets based on network pharmacology and to establish the research foundation for elucidating the mechanism by which berberine ameliorated *S. suis* meningitis in pigs and humans. In addition, we suggest a new approach to manage and avoid streptococcal meningitis by actively pushing for novel veterinary feeds derived from the same food and medicine source and minimizing the use of antibiotics.

## 2. Materials and Methods

**Figure 1:**
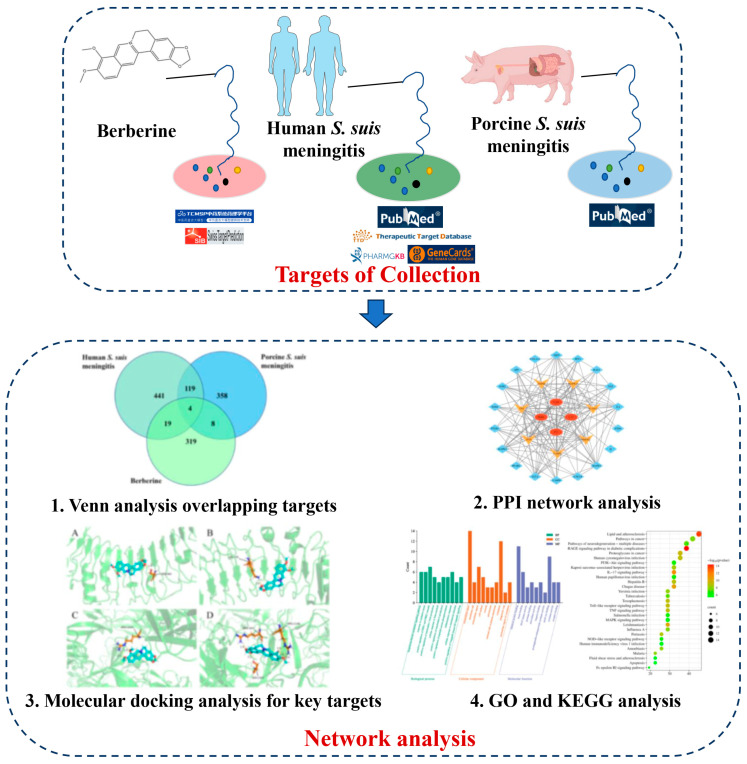
Illustration of the study strategy. (TCMSP means Traditional Chinese Medicine Systems Pharmacology Database).

The study strategy is illustrated in Figure 1.

### 2.1. Screening of Berberine Targets

Berberine targets were obtained from SwissTargetPrediction (http://www.swisstargetprediction.ch) [19] and the Traditional Chinese Medicine Systems Pharmacology Database and Analysis Platform (https://www.tcmsp-e.com) [20]. The targets were supplemented by referring to the literature collected at the National Center for Biotechnology Information (NCBI; https://www.ncbi.nlm.nih.gov). All targets were combined and then deduplicated. Meanwhile, the structure of berberine was imported to admetSAR (http://lmmd.ecust.edu.cn/admetsar2) [21] to predict its pharmacokinetics and toxicity.

### 2.2. Screening of S. suis Meningitis Targets

The PubMed database was searched to identify *S. suis* meningitis targets by using the keywords “porcine *S. suis* meningitis”. Human *S. suis* meningitis targets were collected from GeneCards (https://www.genecards.org) [22], NCBI (https://www.ncbi.nlm.nih.gov), PharmGKB (https://www.pharmgkb.org) [23], and the Therapeutic Target Database (TTD; https://db.idrblab.net/ttd) [24] by using the same keywords. Duplicates were removed to obtain a list of the two lists of potential disease targets.

### 2.3. Prediction of Potential Targets of Berberine in Treating S. suis Meningitis

The list of identified berberine, porcine *S. suis* meningitis, and human *S. suis* meningitis targets were imported to the Venny 2.1 software, which generated a Venn diagram to identify the targets of berberine that could ameliorate *S. suis* meningitis.

### 2.4. Construction of a Protein–Protein Interaction (PPI) Network

The STRING database (https://string-db.org) was used to construct a PPI network of the potential target sets, with the biological species set to *Sus scrofa* and the minimum interaction score set to medium confidence (0.400). The file was saved in the tsv format and imported into the Cytoscape 3.8.0 software [25] for topological analysis. The genes were sorted according to the degree values. Genes with scores greater than the average were selected as the core targets.

### 2.5. Molecular Docking

Molecular docking was performed for the top four critical berberine protein targets to alleviate *S. suis* meningitis in pigs and human. The structure of the active pharmaceutical ingredient was downloaded from the PubChem database and imported into the ChemBio3D 14.0 software for processing; the energy was minimized and saved as a ligand. In the Uniprot database (https://www.uniprot.org), the three-dimensional (3D) structure of the *Sus scrofa* (*S. scrofa*) target protein predicted by AlphaFold and the 3D structure of the *Homo sapiens* target protein download from the Protein Data Bank (https://www.rcsb.org) was exported. The SYBYL-X 2.0 software [26] was used to simulate the ligand–receptor docking, the PyMOL 3.1.3.1 software [27] was used to visualize the model ligand–receptor 3D diagrams, and LigPlot+ v.2.2 software was used to obtain 2D images.

### 2.6. Gene Ontology (GO) and Kyoto Encyclopedia of Genes and Genomes (KEGG) Enrichment Analyses

GO and KEGG enrichment analysis was performed for the potential targets via DAVID (https://david.ncifcrf.gov). The relevant genes were uploaded, the species was set to *S. scrofa*, and the *p*-value was set to <0.05. The enrichment results were processed using bioinformatics (http://www.bioinformatics.com.cn) to obtain histograms and bubble charts.

## 3. Results

### 3.1. Screening of the Potential Berberine Targets in Treating S. suis Meningitis

**Figure 2:**
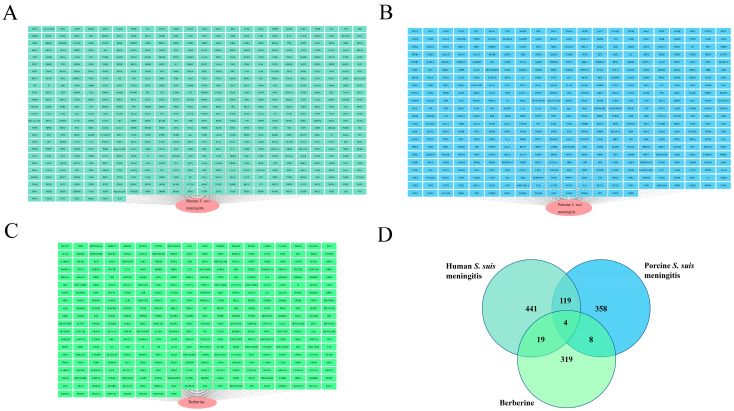
Berberine and streptococcal meningitis targets. (**A**) Human *S. suis* meningitis targets. (**B**) Porcine *S. suis* meningitis targets. (**C**) Berberine targets. (**D**) Venn diagram of the three groups of targets. (Note: green represents berberine targets; blue represents porcine *S. suis* meningitis targets; and Cyan represents Human S. suis meningitis targets; the data details of Figure 2 in Supplementary Materials Tables S1 and S2).

The berberine, porcine *S. suis* meningitis, and human *S. suis* meningitis targets are summarized in Figure 2. We identified 350 berberine targets (Figure 2A), 490 porcine *S. suis* meningitis targets (Figure 2B), and 583 human *S. suis* meningitis targets (Figure 2C). Moreover, the admetSAR results demonstrated that berberine can cross the blood–brain barrier, can be absorbed by the intestine, and do not exert teratogenicity or nephrotoxicity (Table 1).

### 3.2. Screening of Common Targets of Berberine and S. suis Meningitis

The potential target sets are summarized in a Venn diagram (Figure 2D). There are four common porcine *S. suis* meningitis and human *S. suis* meningitis targets, namely, Toll-like receptor 4 (TLR4), fibronectin (FN1), superoxide dismutase1 (SOD1), and catalase (CAT).

### 3.3. Analysis of the PPI Network and Screening of Core Targets

**Figure 3:**
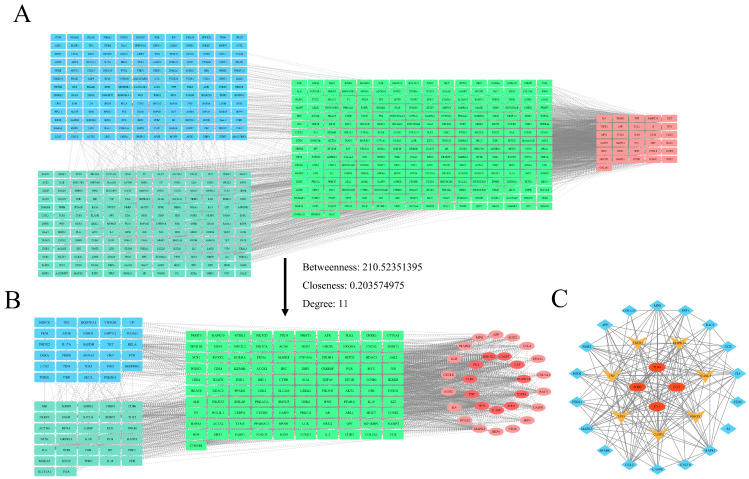
Screening of the core targets of berberine in alleviating meningitis in pigs and humans based on the STRING database and Cytoscape. (**A**) Cytoscape analysis of the interconnection of berberine and *S. suis* meningitis targets (Note: green represents berberine targets; blue represents porcine *S. suis* meningitis targets; and Cyan represents Human *S. suis* meningitis targets.). (**B**) Cytoscape parameters (betweenness > 210.52351395, closeness > 0.203574975, and degree > 11) were set to evaluate the relationship between the key targets (Note: green represents berberine targets; blue represents porcine *S. suis* meningitis targets; and Cyan represents Human *S. suis* meningitis targets.). (**C**) Core targets for further screening based on the Cytoscape database.

We submitted the target proteins to STRING version 11.0 (http://string-db.org) to construct a PPI network, which includes 11 core targets, including TLR4, FN1, SOD1, CAT, tumor necrosis factor (TNF), transforming growth factor beta (TGF-β), tumor protein P53 (TP53), caspase-3 (CASP3), mitogen-associated protein kinase 14 (MAPK14), intercellular adhesion mole9ocule 1 (ICAM-1), and heme oxygenase 1 (HMOX1) (Figure 3). Among them, TLR4, FN1, SOD1, and CAT are the most critical targets; they could represent the targets by which berberine alleviates *S. suis* meningitis.

### 3.4. Molecular Docking Rresults

**Figure 4:**
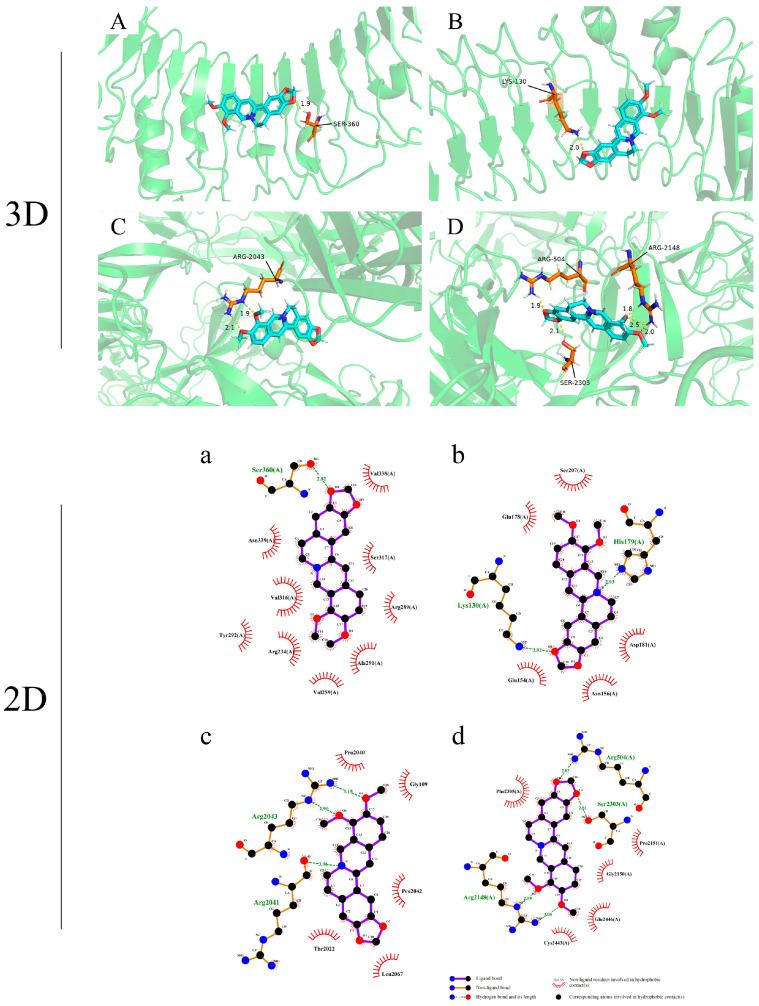
Molecular docking diagram. Molecular models of the binding of berberine with human and porcine TLR4 and FN1. The results are shown as 3D and 2D diagrams: (**A**,**a**) berberine–TLR4 (docking score = 4.17) in humans. (**B**,**b**) Berberine–TLR4 (docking score = 4.19) in pigs. (**C**,**c**) Berberine–FN1 (docking score = 3.78) in humans. (**D**,**d**) Berberine–FN1 (docking score = 4.35) in pigs.

Berberine and the most critical targets TLR4, FN1, SOD1, and CAT were evaluated by molecular docking. The results showed that berberine has a good docking activity to bind the docking pocket of each target protein, and the main forces involved were hydrophobic forces and hydrogen bonds (Figure 4). These interactions might play key roles in the mechanism by which berberine alleviates *S. suis* meningitis. Berberine binds to TLR4 by forming hydrogen bonds with Ser360 (docking score = 4.17) (Figure 4A,a) and Lys130 (docking score = 4.19) (Figure 4B,b) of humans and pigs, respectively. Similarly, berberine is predicted to dock via hydrogen bonds Arg2043 (docking score = 3.78) (Figure 4C,c) and Arg504, Arg2148, and Ser2303 (docking score = 4.35) (Figure 4D,d) in the binding pocket of FN1 of humans and pigs, respectively. However, SOD1 and cat had low docking scores. Thus, berberine may affect its function by competitively inhibiting the binding of the docking pocket to the TLR4 and FN1 target receptors. These interactions might play roles in alleviating *S. suis* meningitis in pigs and humans.

### 3.5. GO and KEGG Enrichment Analysis and the Component–Disease Pathway–Target Network

**Figure 5:**
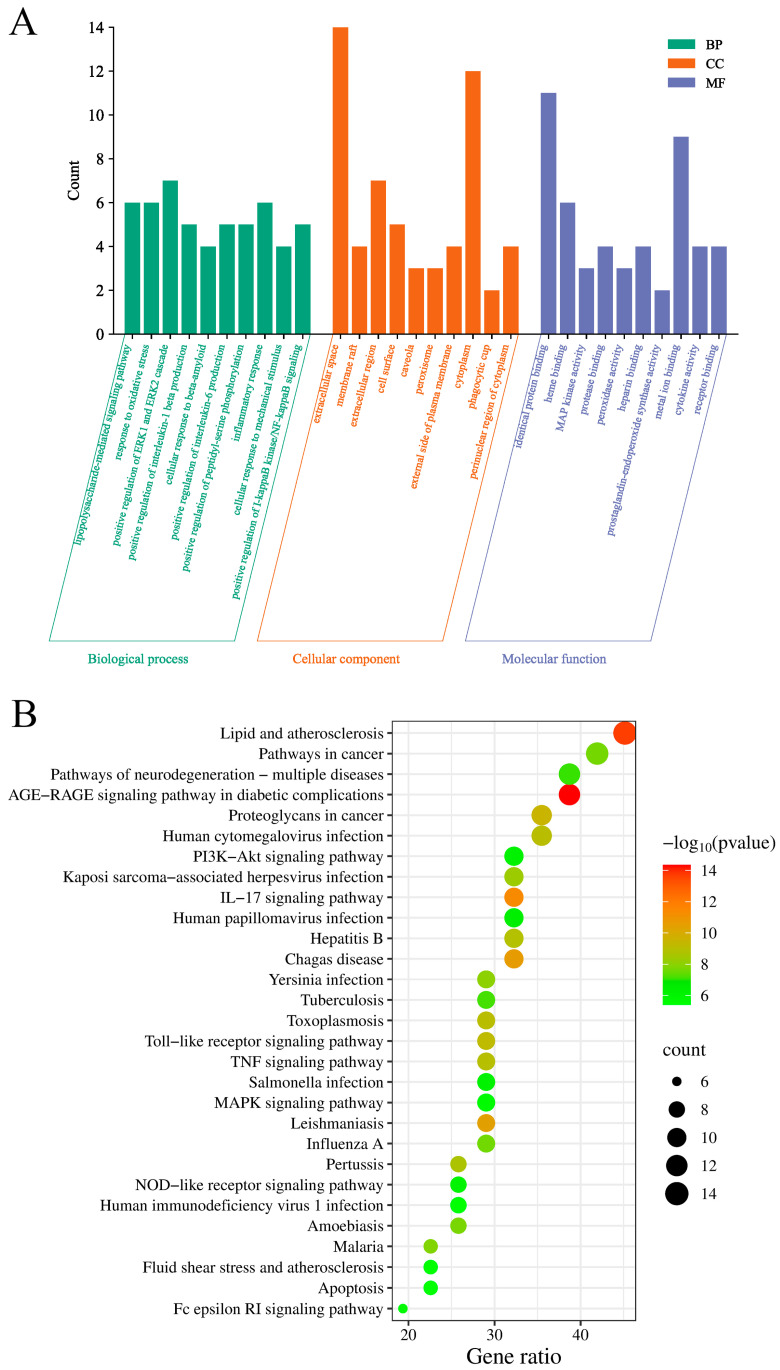
(**A**) GO and (**B**) KEGG enrichment analysis of potential targets of berberine to alleviate *S. suis* meningitis.

We performed GO and KEGG enrichment analyses of the potential targets by using DAVID to clarify the role of core targets in biology and signal transduction pathways. As shown in Figure 5A, GO analysis revealed the enrichment of biological processes such as the positive regulation of the extracellular-signal-regulated kinase 1 (ERK1) and ERK2 cascade, the inflammatory response, and the response to oxidative stress. The KEGG enrichment analysis showed the enrichment of the interleukin 17 (IL-17), phosphoinositide 3-kinase (PI3K)-Akt, TLR, TNF, and MAPK signaling pathways (Figure 5B). Overall, the enrichment of proteins that are closely related to oxidative stress, the inflammatory response, and their related signaling pathways were showed by GO and KEGG analyses. Hence, berberine may alleviate *S. suis* meningitis in pigs and humans by regulating oxidative stress and inflammatory signaling pathways.

## 4. Discussion

Pigs of any age can be infected with streptococcal meningitis, a common disease in the livestock and pig-farming industries. Among them, nursing piglets and weaned piglets are high-risk groups. With the widespread outbreak of streptococcal disease in pigs, there have been increasing reports of human infections and cases of fatalities, posing a serious threat to human health and causing significant economic losses [10,28]. Therefore, it is crucial to strengthen research on *S. suis* meningitis in pigs and humans, to reduce the harm caused by the disease, and to develop effective preventive and therapeutic drugs.

We used network pharmacology methods to predict the functional targets of berberine in the treatment of *S. suis* meningitis in pigs. We screened a total of 31 potential targets, and the PPI network analysis showed that the core targets of berberine include TLR4, FN1, SOD1, CAT, TNF, TGF-β, TP53, CASP3, MAPK14, ICAM-1, and HMOX1, indicating that berberine may exert therapeutic effects on *S. suis* meningitis through multiple targets and pathways. GO and KEGG enrichment analyses revealed that berberine is mainly associated with processes such as the lipopolysaccharide-mediated signaling pathway, oxidative stress, protein binding, kinase activity, extracellular fluid, the cytoplasm, protein binding, and metal ion binding. At the same time, berberine may exert therapeutic effects on *S. suis* meningitis by mediating neurodegenerative signaling pathways and inflammation via the IL-17, PI3K-Akt, TNF, TLR, and MAPK signaling pathways.

TLR4 is a crucial regulatory factor in inflammation. As a pattern recognition receptor, TLR4 is expressed in a range of cells, including microglia, astrocytes, and neurons and plays an important role in regulating the inflammatory response to stress [29,30,31]. Activating TLR4 signaling will lead to the nuclear factor kappa-light-chain-enhancer of activated B cell (NF-κB) expression and the production of the pro-inflammatory cytokines TNF, IL-1β, and IL-6 and the activation of MAPK pathways, leading to pro-inflammatory effects and neuroinflammation in the abovementioned cells [32,33]. In addition, TNF directly drives inflammatory responses by inducing the expression of inflammatory genes and indirectly promotes inflammation and immune responses as well as disease development by inducing cell death and activating the MAPK and NF-κB signaling pathways to promote the expression of inflammatory genes [34].

In addition, damage to the tight connections of the blood–brain barrier is also an important target of *S. suis* meningitis. FN1 exists as a dimer or multimer in plasma, on cell surfaces, and in the extracellular matrix and is involved in processes such as cell migration and adhesion [35,36,37]. It is associated with a poor prognosis in diseases such as glioma, gastric cancer, and thyroid cancer [38,39] and is highly expressed in patients with neuroinflammation and glioblastoma [40,41]. FN1 overexpression can affect the immune microenvironment and cause immune cell infiltration. The loss of FN1 can inhibit the proliferation and migration of glioblastoma cells [42]. These studies indicate that the overexpression of FN1 promotes the development of bacterial infection-induced central nervous system inflammation, suggesting that FN1 may be an important target in bacterial meningitis.

SOD1 is an enzyme with antioxidant activity that maintains the balance between oxidation and antioxidation in the body, protects the normal function of brain cells, and plays a crucial role in clearing excess free radicals [43]. In a mouse model with a 50% reduction in SOD1 protein expression (Sod1+/−), the lower protein levels cause motor dysfunction and increase neurodegeneration and the sensitivity to other cellular stresses such as cerebral ischemia, leading to increased blood–brain barrier permeability [44]. Hence, the sensitivity of neurons to oxidative damage seems to be dependent on the lack of SOD1. HMOX1, a classical antioxidant protein, exerts anti-inflammatory and antioxidant effects when activated [45,46]. Patients with HMOX1 deficiency and mice with Hmox1 gene deletion show increased inflammation and increased susceptibility to oxidative stress [47], indicating that HMOX1 is an important regulator of antioxidant and anti-inflammatory responses.

We speculate that after *S. suis* invades the host, it increases the levels of TLR4, TNF, and FN1, leading to the release of a large amount of superoxide anions and nitric oxide (NO), thus resulting in oxidative stress. These changes would trigger a series of reactions leading to blood–brain barrier disruption, inflammatory damage, and nerve injury. As a result, cells are severely damaged, leading to meningitis. The body may resist *S. suis* meningitis by regulating anti-inflammatory and antioxidant genes such as HMOX1 and SOD1 and inhibiting the expression of inflammatory factors such as TNF.

Continuous in-depth research on traditional Chinese herbs and the active ingredients of Chinese medicine has provided new insights into the treatment of diseases. Berberine, a component of several traditional Chinese herbs, has good anti-inflammatory activity and can reduce the expression of various inflammatory cytokines such as TNF-α, thereby exerting anti-inflammatory effects [48]. Berberine can inhibit TGF-β1, upregulate E-cadherin expression, increase cell adhesion, and decrease cell migration [16]. It can also inhibit the phosphorylation of c-Jun N-terminal kinase (JNK) and NF-κB to suppress the activation of the TAK1/JNK and TAK1/NF-κB signaling pathways, thereby reducing the expression of ICAM-1, inhibiting the expression of TGF-β and some inflammatory factors, such as IL-1β, IL-6, and TNF-α, and alleviating inflammation [49].

Berberine can also increase the activity of SOD and CAT in mice and decrease the level of kidney injury molecule-1 (KIM-1), NO, malondialdehyde (MDA), TNF-α, and IL-1β to reduce oxidative stress and inflammatory effects [50,51]. Similarly, berberine can participate in the TLR4 signaling pathway to alleviate inflammatory reactions, significantly reduce reactive oxygen species levels, enhance the antioxidant defense, induce the expression of Nrf2 and HOMX1 through the PI3K/Akt pathway, and alleviate oxidative stress [52]. Based on these functions, we speculate that berberine regulates the levels of TGF-β, TNF-α, ICAM-1, TLR4, and FN1; induces the expression of anti-inflammatory and antioxidant genes such as HMOX1 and SOD1; regulates signaling pathways related to neurodegeneration (TNF, TLR4, PI3K/Akt, etc.); and alleviates neuroinflammation by modulating multiple pathways. Additionally, berberine could enhance antioxidant and anti-inflammatory levels and mitigate inflammatory damage. Hence, berberine may exert therapeutic effects on *S. suis* meningitis through coordinated actions involving multiple targets and pathways. The mechanisms of action of these targets and signaling pathways require validation to serve as a basis for the clinical application of berberine.

## 5. Conclusions

In summary, we analyzed the targets of berberine in the treatment of *S. suis* meningitis by using network pharmacology. Our findings indicate that berberine exerts its effect on *S. suis* meningitis through multiple targets and pathways, of which anti-inflammatory and antioxidant stress are key roles. Our findings provide a reference for the development of new drugs to treat humans and pigs with *S. suis* meningitis and new types of animal feed and environmentally friendly and antibiotic-free animal-derived food products. These endeavors should contribute to modernizing the farming industry.

## Figures and Tables

**Table 1:** Prediction of ADMET for berberine.

Model Name	Berberine	Model Name	Berberine
Ames mutagenesis	−	Honey bee toxicity	−
Acute Oral Toxicity (c)	III	Hepatotoxicity	+
Androgen receptor binding	+	Human Ether-a-go-go-Related Gene inhibition	−
Aromatase binding	−	Human Intestinal Absorption	+
Avian toxicity	−	Human oral bioavailability	−
Blood–Brain Barrier	+	MATE1 inhibitior	−
BRCP inhibitior	−	Mitochondrial toxicity	+
Biodegradation	−	Micronuclear	+
BSEP inhibitior	+	Nephrotoxicity	−
Caco-2	+	Acute Oral Toxicity	1.588989
Carcinogenicity (binary)	−	OATP1B1 inhibitior	+
Carcinogenicity (trinary)	Non-required	OATP1B3 inhibitior	+
Crustacea aquatic toxicity	+	OATP2B1 inhibitior	−
CYP1A2 inhibition	+	OCT1 inhibitior	+
CYP2C19 inhibition	−	OCT2 inhibitior	−
CYP2C9 inhibition	−	P-glycoprotein inhibitior	+
CYP2C9 substrate	−	P-glycoprotein substrate	−
CYP2D6 inhibition	+	PPAR gamma	+
CYP2D6 substrate	−	Plasma protein binding	0.850641
CYP3A4 inhibition	−	Reproductive toxicity	+
CYP3A4 substrate	+	Respiratory toxicity	+
CYP inhibitory promiscuity	+	skin sensitisation	−
Eye corrosion	−	Subcellular localzation	Mitochondria
Eye irritation	−	Tetrahymena pyriformis	1.632231
Estrogen receptor binding	+	Thyroid receptor binding	+
Fish aquatic toxicity	−	UGT catelyzed	−
Glucocorticoid receptor binding	+	Water solubility	−2.97369

## Data Availability

All data in this article are presented in the article.

## Supplementary Materials

The following are available online at https://www.mdpi.com/article/10.3390/toxics13020138/s1, Table S1: The data details of Figure 2A–C, Table S2: The data details of Figure 2D.

## Abbreviation and Symbols

3D	three-dimensional
CASP3	caspase-3
ERK	extracellular signal-regulated kinase 1
FN1	fibronectin 1
GO	Gene Ontology
HMOX1	heme oxygenase 1
ICAM-1	intercellular adhesion molecule 1
IL-17	interleukin 17
JNK	c-Jun N-terminal kinase
KEGG	Kyoto Encyclopedia of Genes and Genomes
KIM-1	kidney injury molecule-1
MAPK	mitogen-associated protein kinase
MDA	malondialdehyde
MIC	minimum inhibitory concentration
NF-κB	nuclear factor kappa-light-chain-enhancer of activated B cells
NO	nitric oxide
PCR	polymerase chain reaction
PI3K	phosphoinositide 3-kinase
PPI	protein–protein interaction
*S. scrofa*	*Sus scrofa*
*S. suis*	*Streptococcus suis*
TGF-β	transforming growth factor beta
TLR4	Toll-like receptor 4
TNF	tumor necrosis factor
TP53	tumor protein P53
